# Systematic druggable genome‐wide Mendelian randomization identifies therapeutic targets for sarcopenia

**DOI:** 10.1002/jcsm.13479

**Published:** 2024-04-21

**Authors:** Kang‐Fu Yin, Ting Chen, Xiao‐Jing Gu, Wei‐Ming Su, Zheng Jiang, Si‐Jia Lu, Bei Cao, Li‐Yi Chi, Xia Gao, Yong‐Ping Chen

**Affiliations:** ^1^ Department of Neurology, West China Hospital Sichuan University Chengdu China; ^2^ Institute of Brain Science and Brain‐Inspired Technology, West China Hospital Sichuan University Chengdu China; ^3^ Mental Health Center, West China Hospital Sichuan University Chengdu China; ^4^ Department of Respiratory The Fourth People's Hospital of Chengdu, Mental Health Center of Chengdu Chengdu China; ^5^ Department of Neurology First Affiliated Hospital of Air Force Military Medical University Xi'an China; ^6^ Department of Geriatrics Dazhou Central Hospital Dazhou China

**Keywords:** Colocalization, Druggable genes, Mendelian randomization, Sarcopenia

## Abstract

**Background:**

There are no effective pharmacological treatments for sarcopenia. We aim to identify potential therapeutic targets for sarcopenia by integrating various publicly available datasets.

**Methods:**

We integrated druggable genome data, cis‐eQTL/cis‐pQTL from human blood and skeletal muscle tissue, and GWAS summary data of sarcopenia‐related traits to analyse the potential causal relationships between drug target genes and sarcopenia using the Mendelian Randomization (MR) method. Sensitivity analyses and Bayesian colocalization were employed to validate the causal relationships. We also assessed the side effects or additional indications of the identified drug targets using a phenome‐wide MR (Phe‐MR) approach and investigated actionable drugs for target genes using available databases.

**Results:**

MR analysis identified 17 druggable genes with potential causation to sarcopenia in human blood or skeletal muscle tissue. Six of them (*HP*, *HLA‐DRA*, *MAP 3K3*, *MFGE8*, *COL15A1*, and *AURKA*) were further confirmed by Bayesian colocalization (PPH4 > 90%). The up‐regulation of *HP* [higher ALM (beta: 0.012, 95% CI: 0.007–0.018, *P* = 1.2*10^−5^) and higher grip strength (OR: 0.96, 95% CI: 0.94–0.98, *P* = 4.2*10^−5^)], *MAP 3K3* [higher ALM (beta: 0.24, 95% CI: 0.21–0.26, *P* = 1.8*10^−94^), higher grip strength (OR: 0.82, 95% CI: 0.75–0.90, *P* = 2.1*10^−5^), and faster walking pace (beta: 0.03, 95% CI: 0.02–0.05, *P* = 8.5*10^−6^)], and *MFGE8* [higher ALM (muscle eQTL, beta: 0.09, 95% CI: 0.06–0.11, *P* = 6.1*10^−13^; blood pQTL, beta: 0.05, 95% CI: 0.03–0.07, *P* = 3.8*10^−09^)], as well as the down‐regulation of *HLA‐DRA* [lower ALM (beta: ‐0.09, 95% CI: −0.11 to −0.08, *P* = 5.4*10^−36^) and lower grip strength (OR: 1.13, 95% CI: 1.07–1.20, *P* = 1.8*10^−5^)] and *COL15A1* [higher ALM (muscle eQTL, beta: ‐0.07, 95% CI: −0.10 to −0.04, *P* = 3.4*10^−07^; blood pQTL, beta: ‐0.05, 95% CI: −0.06 to −0.03, *P* = 1.6*10^−07^)], decreased the risk of sarcopenia. *AURKA* in blood (beta: ‐0.16, 95% CI: −0.22 to −0.09, *P* = 2.1*10^−06^) and skeletal muscle (beta: 0.03, 95% CI: 0.02 to 0.05, *P* = 5.3*10^−05^) tissues showed an inverse relationship with sarcopenia risk. The Phe‐MR indicated that the six potential therapeutic targets for sarcopenia had no significant adverse effects. Drug repurposing analysis supported zinc supplementation and collagenase *clostridium histolyticum* might be potential therapeutics for sarcopenia by activating *HP* and inhibiting *COL15A1*, respectively.

**Conclusions:**

Our research indicated *MAP 3K3*, *MFGE8*, *COL15A1*, *HP*, and *HLA‐DRA* may serve as promising targets for sarcopenia, while the effectiveness of zinc supplementation and collagenase *clostridium histolyticum* for sarcopenia requires further validation.

## Introduction

Sarcopenia has been recognized as a muscle disease, characterized by age‐related loss of muscle mass and function.^1^ According to the definition of the European Working Group on Sarcopenia in Older People (EWGSOP), 4.6% of men and 7.9% of women with an average age of 67 suffer from sarcopenia.^2^ It is a major cause of frailty, functional decline, increased risk of falls, and premature death in the elderly, all imposing a heavy burden on the healthcare system.^3^ Lifestyle measures, such as dietary intervention and physical activity, were recommended as the primary preventive and therapeutic strategies in managing sarcopenia, but such effect is limited.^4^ In addition, therapeutic targets for sarcopenia, which mainly are hormone‐based drugs, such as testosterone and selective androgen receptor modulators, have notable adverse reactions.^5^ Therefore, it is urgent and necessary to explore the pathogenesis of sarcopenia and identify new drug targets.

Recently, the advent of multi‐omics research, which amalgamates data from diverse biological domains such as transcriptomics and proteomics, facilitates cross‐validation among different components and provides robust evidence for the discovery of potential biomarkers and therapeutic targets.^6^ Expression quantitative trait Loci (eQTL) and protein quantitative trait loci (pQTL), combined with the analysis of Genome‐Wide Association Study (GWAS) datasets, can aid in the identification of disease‐related genetic variations at both gene expression^7^ and protein levels.^8^ Notably, cis‐acting variants, genetic tools proximal to the transcription gene unit and located on the same chromosome as the transcription gene, can more readily identify phenotype‐related functional variations, thereby directly revealing the genetic mechanisms governing gene expression regulation.^9^, ^10^

Identifying the appropriate drug targets for a disease is the pivotal first step in drug development, and the concept of the ‘Druggable genome’ has dramatically facilitated the discovery of repurposed drug targets.^11^ The ‘Druggable genome’ refers to a subset of the genome that encodes genes with potential drug targets. These genes typically encode proteins with drug‐binding sites, which enhance the success rate of drug target discovery.^12^ Furthermore, research on the ‘Druggable genome’ can expedite the drug development process, providing more information and guidance for drug design.^11^ Mendelian randomization (MR) is a method that employs genetic variations closely associated with exposure factors as instrumental variables (IVs) to assess the causal effect of exposure on outcomes, providing compelling robustness in causal inference.^13^ The research strategy of MR analysis by combining the ‘Druggable genome’ and cis‐eQTL/cis‐pQTL has identified potential drug targets for numerous diseases, such as stroke and Alzheimer's disease.^14^, ^15^

Several studies have examined genetic and proteomic changes that could impact sarcopenia; however, only a limited number have utilized methodologies like MR/Steiger tests to investigate the causal links between these changes and sarcopenia.^16^, ^17^ However, a systematic establishment of drug targets specifically for sarcopenia is yet to be achieved, such as lack of cross‐validation among different components (pQTL, eQTL, plasm, and skeletal muscle) and the application of ‘Druggable genome’.^16^, ^17^ In this research, we aim to identify effective drug targets for sarcopenia by integrating druggable genome data, transcriptomics data, proteomics data, and GWAS summary data of three sarcopenia‐related traits.

## Methods

**Figure 1:**
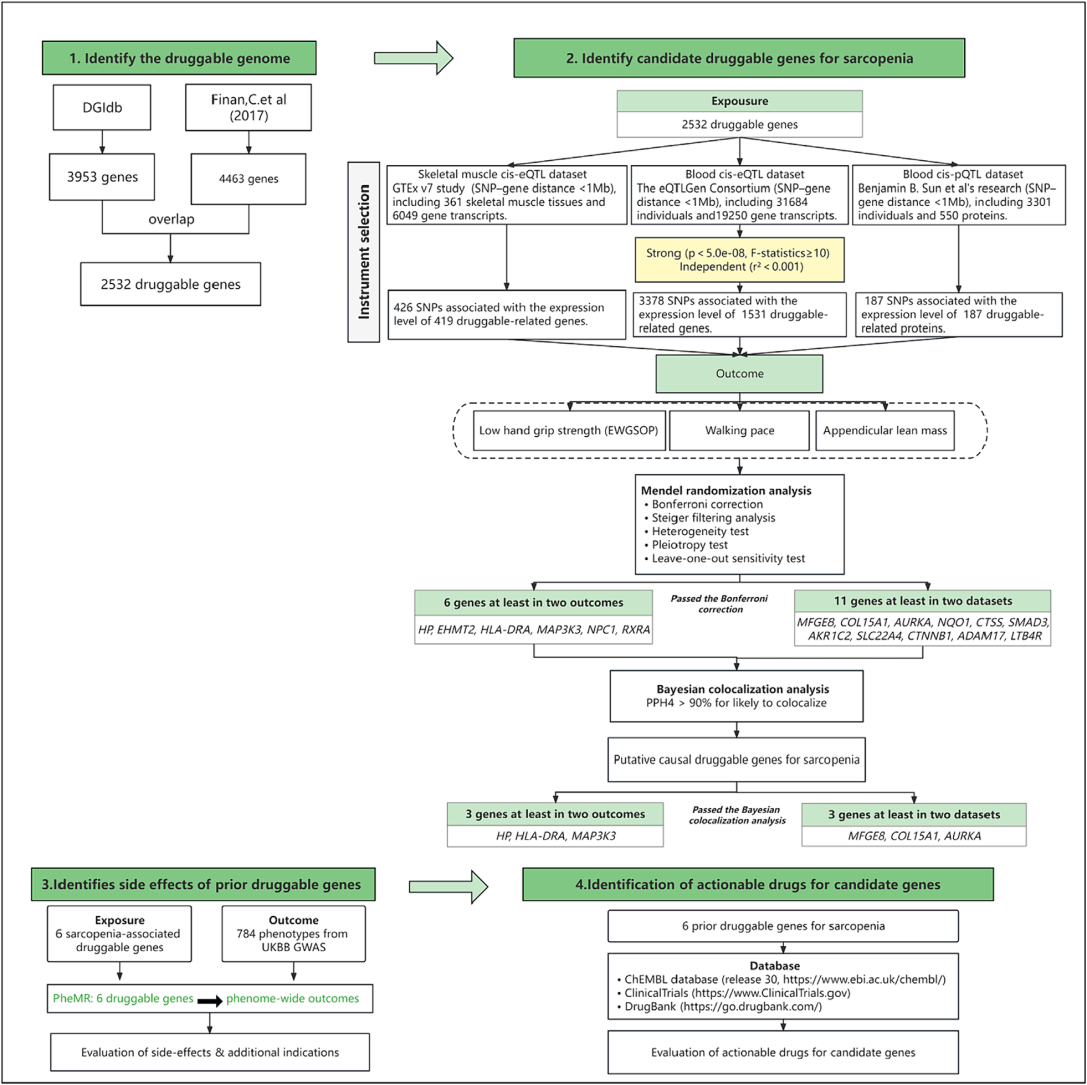
Flow diagram of the study. Initially, we obtained 2532 known druggable genes from the DGIdb database^18^ and the research of Finan et al.^11^ subsequently, we utilized eQTL/pQTL data from human blood and skeletal muscle tissues to construct an eQTL/pQTL tool for druggable genes, filtering out independent genetic variants significantly associated with druggable gene expression (serving as IVs), which are located within a 1 Mb range upstream and downstream of the coding sequence (cis). In the MR analysis, we preliminarily identified potential causal genetic variants for sarcopenia using these IVs. We also employed Bayesian colocalization methods to detect shared causal genetic variants. Finally, we assessed the potential side effects or additional indications of the identified priority druggable genes through a Phe‐MR in UK biobank GWASs,^35^ and researched their druggability and clinical development capabilities in relevant databases or websites. eQTL, expression quantitative trait loci; eQTLGen consortium, expression quantitative trait loci generation consortium; GTEx, genotype‐tissue expression; pQTL, protein quantitative trait lociDGIdb, drug‐gene interaction database; Phe‐MR, phenome‐wide Mendelian randomization analysis.

The conceptual diagram of the current study is shown in Figure 1. The datasets used were summary data; all informed consent and ethical approval were obtained in the original studies.

### Data sources

#### Identification of druggable genes

The druggable genes in our study were derived from the Drug‐Gene Interaction Database (DGIdb v4.2.0, https://www.dgidb.org/downloads)^18^ and the research report by Finan. C et al.^11^ (Table 1, Tables S1 and S2). DGIdb is an online resource that provides information on drug‐gene interactions from publications, databases, and other web‐based sources.^18^ We downloaded the Interactions data (released in February 2022) from DGIdb, which includes all druggable genes with drug‐gene interactions mapped to Entrez genes. Additionally, the druggable gene reported by Finan et al. was also incorporated into our study because it connected GWAS‐identified loci related to complex diseases with druggable genes, thus facilitating the identification and validation of drug targets.^11^

**Table 1:** Data sources for the MR analysis in the current study

Type of dataset	Data subtype	Source	Sample size^a^	Population	Download site
Druggable genome	DGIdb 4.0	Freshour.SL, et al. 2020	‐	‐	https://www.dgidb.org/downloads.
	Prior druggable gene	Finan.C, et al. 2017	‐	‐	Finan C, et al. PMID: 28356508.
QTL datasets	Skeletal muscle cis‐eQTL	GTEx Consortium (Aguet F, et al. 2017)	361	European	https://gtexportal.org/home/datasets.
	Blood cis‐eQTL	eQTLGen Consortium (Võsa U, et al. 2021)	31 684	European	https://www.eqtlgen.org/cis‐eqtls.html.
	Blood cis‐pQTL	Sun BB, et al. 2018	3301	European	https://www.ebi.ac.uk/gwas/downloads/summary‐statistics.
GWAS summary	LOW hand grip strength	UK Biobank (Jones G, et al. 2021)	Cases: 48 596, Controls: 207 927	European	https://gwas.mrcieu.ac.uk/datasets. GWAS ID (ebi‐a‐GCST90007526)
	Walking pace	UK Biobank (Ben Elsworth, et al. 2018)	459 915	European	https://gwas.mrcieu.ac.uk/datasets. GWAS ID (ukb‐b‐4711)
	Appendicular lean mass	UK Biobank (Pei YF, et al. 2020)	450 243	European	https://gwas.mrcieu.ac.uk/datasets. GWAS ID (ebi‐a‐GCST90000025)
	784 Phenotypes	UK Biobank (Zhou, et al. 2018)	408 961	European	https://www.leelabsg.org/resources.

DGIdb, drug‐gene interaction database; eQTL, expression quantitative trait loci; eQTLGen Consortium, expression quantitative trait loci generation consortium; GTEx, genotype‐tissue expression; GWAS, genome‐wide association study; pQTL, protein quantitative trait loci.

^a^ Sample size shown as a total number for quantitative traits, continuous traits and cases/controls for binary traits.

#### eQTL and pQTL datasets

Due to the consideration that cis‐regulatory elements have more direct and specific biological effects compared to trans‐regulatory elements,^19^ we utilized cis‐eQTL/cis‐pQTL data from human blood and cis‐eQTL data from skeletal muscle tissue (genetic variants within a 1 Mb range on either side of the coding sequence in druggable genes) (Table 1). The blood cis‐eQTL data, containing information on 19 250 gene transcripts from 31 684 individuals, were obtained from the eQTLGen Consortium.^20^ Blood cis‐pQTL data were derived from the report by Sun et al., encompassing 550 protein information from 3301 individuals.^21^ Additionally, we acquired skeletal muscle tissue cis‐eQTL data from the genotype‐tissue expression (GTEx) Consortium, which included information on 6049 gene transcripts from 361 individuals.^22^

#### GWAS datasets for three sarcopenia‐related traits

According to the 2018 EWGSOP definition of sarcopenia, emphasis is placed on diminished muscle strength (exemplified by low hand grip strength) as a pivotal feature of the condition.^23^ Additionally, the diagnosis is ascertained by assessing decreased muscle mass and quality [represented by appendicular lean mass (ALM)], and impaired physical performance (indicated by walking pace) serves as a marker of severe sarcopenia.^23^ Consequently, we obtained GWAS summary data for low hand grip strength, derived from a meta‐analysis of 256 523 Europeans aged ≥60 years across 22 cohorts,^24^ and we also collected GWAS summary data for ALM in 450 243 individuals^25^ and walking pace in 459 915 individuals from the UK Biobank^26^ (Table 1).

### Instrument selection

The MR method utilizes single nucleotide polymorphisms (SNPs) closely associated with exposure as instrumental variables (IVs) to assess the causal effect of exposure on outcomes. Eligible IVs must satisfy three assumptions^27^: (1) IVs are directly related to exposure; (2) IVs are independent of any confounding factors; and (3) IVs should be independent of the outcome. First, we perform a cross‐analysis of 2532 overlap potential druggable genes with human blood eQTL/pQTL and skeletal muscle eQTL datasets to obtain the eQTL/pQTL dataset for druggable genes (Figure 1). Next, we extract genetic variants (cis) closely related to druggable gene expression, located within a 1 Mb range surrounding the druggable gene coding sequence. Then, to minimize the impact of pleiotropy and not violate the three MR assumptions—that IVs should only affect the outcome through their impact on exposure and not via other pathways, we adopt a genome‐wide significance threshold (*P* < 5*10^−8^) and an F‐statistic ≥10 to obtain strong IVs.^28^ Finally, to ensure that each selected significant SNP is independent and to exclude the influence of pleiotropy due to linkage disequilibrium (LD), we set the LD coefficient *r* ^2^ to 0.001 and the LD window width to 10 Mb and use the clumping function of the Two‐Sample MR package to obtain IVs.^29^

### Mendelian randomization and Steiger filtering analysis

Upon determining the IVs, we extracted effect estimates for the same variants or their proxies in the sarcopenia GWAS dataset for data harmonization. We employed the Wald ratio or inverse‐variance weighted (IVW) method under a random‐effects model (REM) to estimate the association between exposure and outcome. The *P*‐value of druggable genes was Bonferroni‐corrected (Bonferroni threshold: skeletal muscle eQTL at 0.05/419, blood eQTL at 0.05/1531, and blood pQTL at 0.05/187, respectively). Additionally, we conducted sensitivity analyses using MR Egger, weighted mode, and weighted median.^30^ Heterogeneity was quantified by the IVW Q statistic, and pleiotropy was assessed by the MR‐Egger intercept.^31^ We employed the MR‐PRESSO global test to detect outliers, and if significant SNP outliers were detected, they were removed from the analysis.^32^ Uncorrected *P* < 0.05 was considered significant in heterogeneity and pleiotropy analyses. Finally, causal directionality was evaluated by the Steiger analysis method.^33^ The Steiger filtering method was applied using the ‘TwoSampleMR’ R package to ensure the results were not distorted by the presence of reverse causation. Results are presented as a categorical variable to aid comprehension: true if the effect direction is from exposure to outcome at *P* < 0.05; false if reversed at *P* < 0.05; uncertain if *P* ≥ 0.05. All data analyses were performed using R software (version 4.1.2) and R packages (TwoSampleMR, MR‐PRESSO, and Rmediation).

### Bayesian colocalization and protein–protein interaction (PPI)

Bayesian colocalization is a statistical method to investigate whether the observed association signals in two traits (Trait 1: potential druggable genes, Trait 2: sarcopenia‐related traits) originate from the same genetic variant. This is determined by calculating the posterior probabilities of five hypotheses^34^: (i) PPH0, no association for both traits; (ii) PPH1, the association only for Trait 1 with the genetic variant; (iii) PPH2, the association only for Trait 2 with the genetic variant; (iv) PPH3, both traits are associated with the genetic variant, but the associations are caused by different genetic variants; (v) PPH4, both traits are associated with the genetic variant, and the associations are caused by the same genetic variant. If the posterior probability PPH4 > 90%, it is considered that potential druggable genes and sarcopenia share the same genetic variant.^34^ We performed Bayesian analysis using the ‘coloc’ package in R (http://cran.r‐project.org/web/packages/coloc).

To investigate the interactions among druggable genes prioritized through MR and further validate their involvement in sarcopenia pathogenesis, we conducted PPI analyses on the druggable genes identified before and after Bayesian analysis. The PPI networks were constructed using the STRING database (version 12.0, available at https://string‐db.org/).

### Phenome‐wide MR analysis

The objective of our phenome‐wide MR (Phe‐MR analysis) analysis was to determine causal relationships between the identified druggable genes and other disease traits to assess their potential associated side effects or additional indications. Zhou et al. employed the SAIGE (Scalable and Accurate Implementation of Generalized Mixed Models) method to analyse over 1400 binary phenotypic samples from 408 961 European ancestry British white participants in the UK Biobank.^35^ As our previous study,^14^ we obtained 784 non‐sarcopenia diseases or traits with a sample size of more than 500 from the SAIGE GWAS (https://www.leelabsg.org/resource) to ensure statistical validity (Table 1 and Table S12).

### Identification of actionable drugs for candidate genes

We searched the DrugBank database (version 5.1.10, https://go.drugbank.com), ChEMBL database (version 33, https://www.ebi.ac.uk/chembl), and ClinicalTrials (https://www.clinicaltrials.gov) to evaluate the drug development for the identified druggable genes, including obtaining drug molecule types, indications for drug targets, and clinical development activities.

## Results

### Druggable genome

We downloaded 3953 potential drug target genes from DGIdb v4.2.0 (Table S1)^18^ and extracted 4463 druggable genes from Finan et al.'s study (Table S2).^11^ To ensure the selected druggable genes were both reliable and had a higher likelihood of being effective drug targets, we further analysed 2532 druggable genes that were validated by both sources and possessed official names assigned by the Human Genome Nomenclature Committee (Table S3).

### Candidate druggable genes for sarcopenia‐related traits

We overlapped 2532 potential druggable genes with genes in the human blood and skeletal muscle eQTL/pQTL datasets, then extracted genetic variations within a 1 Mb range on either side of the coding sequences of the overlapping druggable genes. After IVs selection and quality control, we obtained 426 SNPs associated with 419 druggable genes from the human skeletal muscle tissue cis‐eQTL, 3378 SNPs associated with 1531 druggable genes from the human blood cis‐eQTL, and 187 SNPs associated with 187 druggable genes from the human blood cis‐pQTL as IVs representing exposure for MR analysis (Figure 1 and Table S4).

Next, we conducted MR analysis on exposure with three GWAS summary datasets of sarcopenia‐related traits. We found 205, 393, and 79 druggable genes in skeletal muscle eQTL, human blood eQTL, and human blood pQTL, respectively, have a potential causal relationship with sarcopenia (at least one outcome out of three sarcopenia‐related traits) (*P* <0.05, Tables S5–S7). After multiple corrections, we found that 21 druggable genes in the human blood pQTL (Bonferroni‐corrected *P* < 0.05/187, Table S8), 63 druggable genes in the human blood eQTL (Bonferroni‐corrected *P* < 0.05/1531, Table S9), and 67 druggable genes in the human skeletal muscle eQTL (Bonferroni‐corrected *P* < 0.05/419, Table S10) have a causal relationship with sarcopenia. To make the results more accurate and convincing, we chose the druggable genes that exist in at least two QTL datasets or at least two sarcopenia‐related traits as potential drug targets for sarcopenia. Finally, we found that six druggable genes (*HP*, *EHMT2*, *HLA‐DRA*, *MAP 3K3*, *NPC1*, and *RXRA*) have a causal relationship with two or more sarcopenia‐related traits; 11 druggable genes (*MFGE8*, *COL15A1*, *AURKA*, *NQO1*, *CTSS*, *SMAD3*, *AKR1C2*, *SLC22A4*, *CTNNB1*, *ADAM17*, and *LTB4R*) have a causal relationship with sarcopenia from two or more QTL datasets (Table S11 and Figure S1).

**Figure 2:**
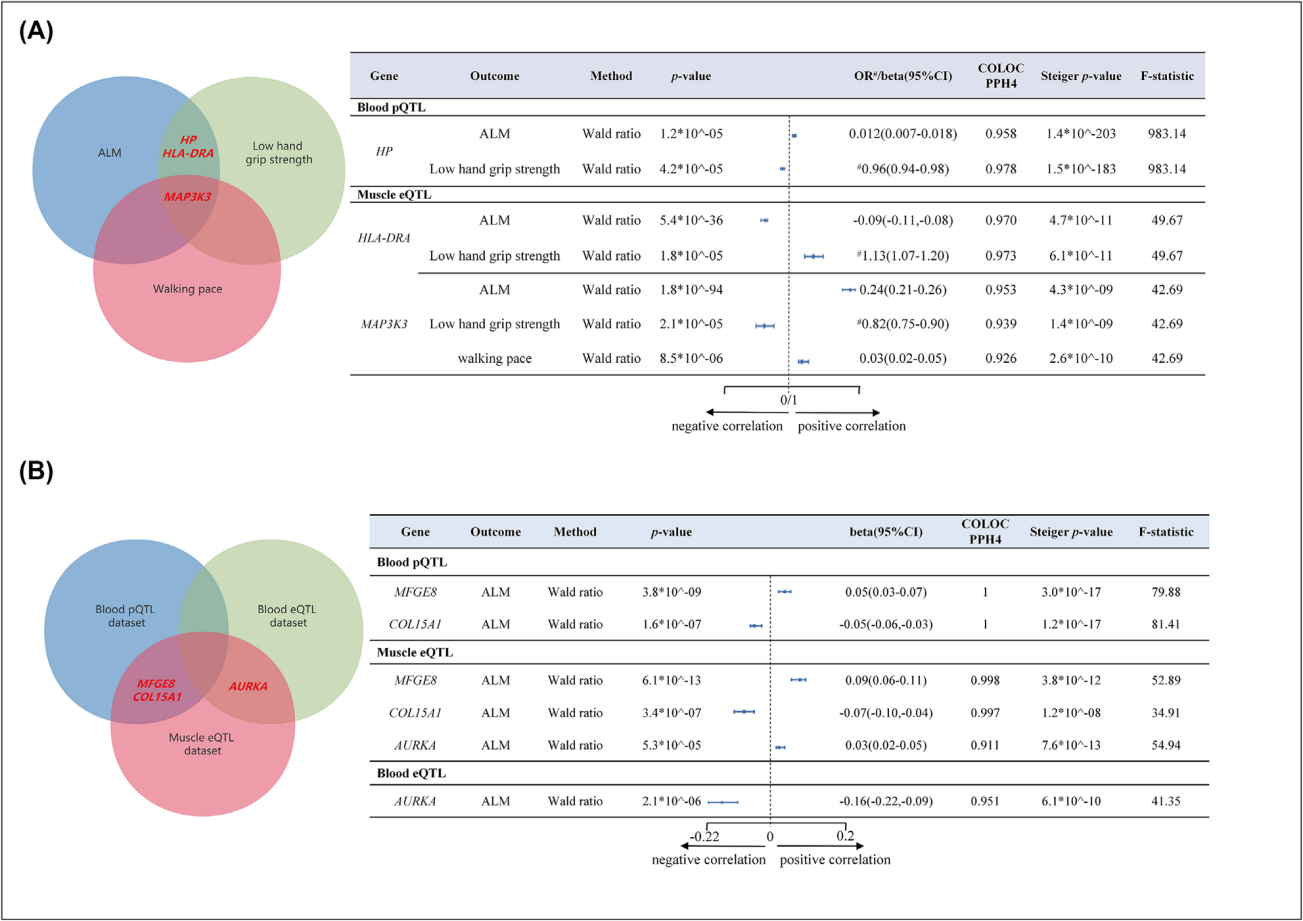
Summary of causal druggable genes for sarcopenia identified through MR and Bayesian colocalization analysis (at least in two outcomes or datasets). To ensure effective IVs and mitigate pleiotropy, we primarily used single SNPs as our tools for exposure. The figure denotes a Steiger *P*‐value < 0.05 as ‘true’, supporting our hypothesis that our IVs cause the outcome variables. Panel (A) shows that three druggable genes passed both Mendelian randomization and Bayesian colocalization analysis and are simultaneously present in two outcomes. Panel (B) shows that three druggable genes passed both Mendelian randomization and Bayesian colocalization analysis and are simultaneously present in two datasets. If the posterior probability of PPH4 is >90%, it is considered that the QTL dataset and sarcopenia share the same variant. ‘^**#**^’ shows that the outcome (low hand grip strength) is binary traits, and the association strength between exposure and outcome is represented by the odds ratio (OR). OR value definition: If OR > 1, exposure may promote the outcome; if OR < 1, exposure may inhibit the outcome. Beta value definition: positive values suggest exposure may promote the outcome, negative values suggest it may inhibit it. ALM, appendicular lean mass.

To identify candidate drug targets with shared genetic variation with sarcopenia‐related traits, we conducted a Bayesian colocalization analysis to verify whether the causal signals observed in the MR analysis originated from the same genetic variation. The results revealed that six potential druggable genes (*HP*, *HLA‐DRA*, *MAP 3K3*, *MFGE8*, *COL15A1*, and *AURKA*) passed the colocalization analysis and were present in more than one QTL datasets or sarcopenia‐related traits (Figure 2 and Table S11). Among them, *HP*, *HLA‐DRA*, and *MAP 3K3* were related to at least two sarcopenia‐related traits (Figure 2A). The abundance of *HP* protein (per 1 SD increase) was associated with higher ALM (beta: 0.012, 95% CI: 0.007–0.018, *P* = 1.2*10^−5^) and higher grip strength (OR: 0.96, 95% CI: 0.94–0.98, *P* = 4.2*10^−5^), indicating the reduced risk of sarcopenia. The transcription level of *HLA‐DRA* in skeletal muscle tissue (per 1 SD increase) was associated with lower ALM (beta: −0.09, 95% CI: −0.11 to −0.08, *P* = 5.4*10^−36^) and lower grip strength (OR: 1.13, 95% CI: 1.07–1.20, *P* = 1.8*10^−5^), indicating the increased risk of sarcopenia. Interestingly, the transcription level of *MAP 3K3* in skeletal muscle tissue (per 1 SD increase) was related to all three sarcopenia‐related traits: higher ALM (beta: 0.24, 95% CI: 0.21–0.26, *P* = 1.8*10^−94^), higher grip strength (OR: 0.82, 95% CI: 0.75–0.90, *P* = 2.1*10^−5^), and faster walking pace (beta: 0.03, 95% CI: 0.02–0.05, *P* = 8.5*10^−6^), indicating the reduced risk of sarcopenia. In addition, *MFGE8*, *COL15A1*, and *AURKA* were validated in different QTL datasets (Figure 2B). The transcription levels of *MFGE8* (per 1 SD increase) and *COL15A1* (per 1 SD decrease) in skeletal muscle tissue eQTL dataset were associated with higher ALM (*MFGE8*, beta: 0.09, 95% CI: 0.06–0.11; *COL15A1*, beta: −0.07, 95% CI: −0.10 to −0.04) and were replicated in the blood pQTL dataset (*MFGE8*, beta: 0.05, 95% CI: 0.03–0.07; *COL15A1*, beta: −0.05, 95% CI: −0.06 to −0.03), indicating the reduced risk of sarcopenia. However, the relationship between the transcription level of *AURKA* in skeletal muscle and blood tissues (per 1 SD increase) and ALM showed opposite results. In our study, our data passed the Steiger filter test, supporting our hypothesis that our IVs cause the outcome variables, not the other way around (Figure 2).

Furthermore, we conducted PPI analyses on the druggable genes identified both before and after Bayesian analysis to investigate their interactions. These interactions were filtered using a medium confidence threshold of 0.4 for the interaction score. Initially, PPI results for the 17 druggable genes identified prior to Bayesian analysis revealed certain gene–gene interactions. Notably, CTSS and CTNNB1 were found to be central in the network, suggesting a potential key role in the pathophysiology of sarcopenia (Figure S2A). Subsequently, PPI analysis was conducted on the six druggable genes identified following Bayesian analysis. However, this analysis did not reveal any significant interactions among these genes (Figure S2B).

### Phe‐MR analysis of sarcopenia‐associated candidate druggable genes

We conducted a Phe‐MR analysis on 784 diseases and traits in the UK Biobank (Table S12). The IVs used for Phe‐MR analysis were consistent with those previously identifying sarcopenia‐related traits druggable genes (nine SNPs for the six druggable genes, see Table S13). Causal effects in the Phe‐MR analysis were considered statistically significant at *P* ≤ 7.08*10^−6^ (Bonferroni‐adjusted: 0.05/9/784). Overall, we found that the increased protein abundance of HP is associated with a reduced risk of disorder of lipoid metabolism (OR: 0.94), especially hyperlipidaemia (OR: 0.94), while the up‐regulation of *HLA‐DRA* is associated with an increased risk of the disorder of lipoid metabolism (OR: 1.17), hyperlipidaemia (OR: 1.17), hypercholesteraemia (OR: 1.17), haematuria (OR: 1.65), chronic sinusitis (OR: 1.27), and inflammatory spondylopathy (OR:2.02) (*P* ≤ 7.08*10^−6^) (Figure S3). Interestingly, the impact direction of increased HP and down‐regulated *HLA‐DRA* is the same as that on sarcopenia, implying that drug targets on HP and HLA‐DRA for sarcopenia may also be beneficial for these diseases, including sarcopenia and dyslipidaemia diseases (Figure S3). However, the other four genes (*MFGE8*, *MAP 3K3*, *COL15A1*, and *AURKA*) are not associated with the 784 diseases and traits (Table S13), indicating that drugs targeted on these genes have no potential adverse effects.

### Identification of actionable drugs for target genes

We have evaluated the pre‐clinical or clinical development activities of six candidate druggable genes for sarcopenia (Table 2). Except for *MFGE8*, formulations related to the other five genes have been evaluated in clinical trials for other diseases, but none have yet been used for the treatment of sarcopenia. Interestingly, zinc chloride and zinc sulfate, serve as activators of the *HP* gene, and ensure the normal expression of the *HP* gene.^36^ And collagenase *clostridium histolyticum* , a binding agent, serve as an inhibitor of *COL15A1* gene expression.^37^, ^38^ These two drugs might be potential therapeutics for sarcopenia by activating *HP* and inhibiting *COL15A1*, respectively. However, Fostamatinib, an inhibitor of *MAP 3K3* and *AURKA*, and coccidioides immitis spherule, an *HLA‐DRA* binding agent, might be not ideal drugs due to the opposite effect for sarcopenia.

**Table 2:** Identification of actionable drugs for target genes

Druggable gene	Molecule type	Compounds	Action type	Clinical development activities	Druggable gene molecular function
MAP 3K3	Small molecule; protein	Fostamatinib	Inhibitor	Fostamatinib^S20^: Approved, investigational. For the treatment of RA and ITP. For controlling ARDS in patients with severe COVID‐19.	ATP binding /MAP kinase kinase kinase activity /metal ion binding/ protein kinase activity/protein serine/threonine kinase activity
MFGE8	Small molecule	‐	‐	‐	Maintenance of intestinal epithelial homeostasis and the promotion of mucosal healing. Promotes VEGF‐dependent neovascularization (by similarity). Contributes to phagocytic removal of apoptotic cells in many tissues.
COL15A1	Small molecule	Collagenase *clostridium histolyticum*	Binder	Collagenase *clostridium histolyticum* ^S21^: Approved, investigational. For promoting debridement of necrotic tissue in burns and skin ulcers as well as to treat Dupuytren's contracture and Peyronie's disease.	Structural protein that stabilizes microvessels and muscle cells, both in heart and in skeletal muscle. Restin potently inhibits angiogenesis.
AURKA	Small molecule	Alisertib; Fostamatinib	Inhibitor, regulator	Alisertib^S22^: For the treatment of various forms of cancer. Fostamatinib^S20^: Approved, investigational. For the treatment of RA and ITP. For controlling ARDS in patients with severe COVID‐19.	ATP binding /histone serine kinase activity / protein kinase activity / protein kinase binding/ protein serine/threonine kinase activity/protein serine/threonine/tyrosine kinase activity
HP	Small molecule	Zinc chloride; Zinc sulfate, unspecified form	Binder	Zinc chloride^S23^: Approved, investigational. For treating zinc deficiencies and associated symptoms and also in total parenteral nutrition. Zinc sulfate, unspecified form^S23^: Approved, experimental. A zinc supplement indicated in parenteral nutrition.	Antioxidant activity/haemoglobin binding/serine‐type endopeptidase activity
HLA‐DRA	Small molecule	ID09C3; Coccidioides immitis spherule	Binder	1D09C3^S24^: Investigational. A monoclonal antibody against lymphoid cancers, is an anti‐MHC class II monoclonal antibody. *Coccidioides immitis* spherule^S25^: Approved. A skin antigen test for delayed‐type hypersensitivity to *Coccidioides immitis*.	MHC class II protein complex binding /MHC class II receptor activity/ peptide antigen binding/polysaccharide binding

The references for the mechanisms of drug action are outlined in Tables S20–S25.

ARDS, acute respiratory distress syndrome; ITP, immune thrombocytopenic purpura; MHC, major histocompatibility complex; RA, rheumatoid arthritis.

## Discussion

Based on these integrated datasets, our study provides potential evidence for the genetic colocalization of six drug target genes (*HP*, *HLA‐DRA*, *MAP 3K3*, *MFGE8*, *COL15A1*, and *AURKA*) with sarcopenia. The phe‐MR analysis did not identify potential adverse effects for these six drug target genes. Upon evaluating the clinical development potential of these drug target genes, we found zinc supplementation and collagenase *clostridium histolyticum* might be potential therapeutics for sarcopenia by activating *HP* and inhibiting *COL15A1*, respectively.

Haptoglobin (HP) is a protein encoded by the *HP* gene that binds with free haemoglobin (HB) released by erythrocytes, with its primary function being related to the clearance of HB in circulation.^39^ When erythrocytes rupture, the released HB can generate free radicals through the Fenton reaction, triggering oxidative stress and causing cellular damage.^40^ However, when HP binds with HB, the complex can be recognized and cleared by hepatic macrophages, thereby preventing oxidative damage caused by HB.^S1^ Within macrophages, the degradation of HB releases iron, facilitating its recycling and reuse, preventing excessive iron accumulation.^S1^ HP can also act as an anti‐inflammatory regulator or pro‐inflammatory activator, inhibiting the proliferation of T cells and regulating the balance between Th1 and Th2 cells.^39^ Our MR studies suggest that increasing the abundance of HP protein may reduce the risk of sarcopenia. Importantly, our Phe‐MR analysis revealed no significant adverse effects associated with the abundance of HP protein. Instead, we found that elevated levels of HP protein may be helpful in reducing the risk of dyslipidaemia‐related diseases (Figure S3). Although the relationship between HP and sarcopenia is not yet clear, research has found that muscle cells in patients with sarcopenia have increased levels of oxidative stress and weakened antioxidant defence mechanisms.^S2^ Therefore, controlling oxidative stress and enhancing antioxidant defences may be an important strategy in the treatment of sarcopenia. Currently, the clinical activators of the *HP* gene mainly consist of zinc supplements, such as zinc chloride and zinc sulfate, for treating zinc deficiency and parenteral nutrition.^36^ However, the current MR analysis does not establish the most effective timing for administering zinc supplements, nor does it address the duration of supplementation. These inquiries warrant further exploration through prospective studies and randomized controlled trials. Although zinc may potentially contribute to the prevention or management of sarcopenia, its ability to reverse the condition requires validation through additional research.

The *COL15A1* gene has been identified as one of the genes with shared genetic co‐localization evidence in relation to sarcopenia, and the increased COL15A1 is a risk factor for sarcopenia. The type XV collagen protein encoded by the *COL15A1* gene is a crucial component of the extracellular matrix, playing a pivotal role in maintaining cell stability and structure.^S3,S4^ However, research has discovered that mutations in the *COL15A1* gene may affect the normal synthesis and function of type XV collagen protein, consequently disrupting the structure of the extracellular matrix. This disruption may impair the normal function of muscle cells, leading to pathological changes in muscles, including muscle cell atrophy, weakened muscle strength, and potentially resulting in the development of muscle diseases such as sarcopenia.^S3, S4^ Currently, COL15A1 formulations have been approved for clinical use, such as collagenase *Clostridium histolyticum* , which has received FDA approval for the removal of necrotic tissue in burns and skin ulcers, as well as the treatment of Dupuytren's contracture and Peyronie's disease^37 38^. Theoretically, inhibiting the COL15A1 is a potential therapeutics for sarcopenia. However, whether collagenase *Clostridium histolyticum* was effectiveness for the treatment of sarcopenia requires clinical trial validation.

The *HLA‐DRA* gene is a part of the human major histocompatibility complex class II molecules, primarily involved in immune responses.^S5^ Research on HLA‐DRA has mainly focused on its association with various autoimmune diseases, such as RA and systemic lupus erythematosus.^S6, S7^ HLA‐DRA functions by presenting antigenic peptide fragments to CD4+T cells, thereby activating immune responses.^S5^ Our MR study revealed that down‐regulation of the *HLA‐DRA* gene may reduce the risk of sarcopenia. Furthermore, based on the results of Phe‐MR analysis (Figure S3), it is suggested that lower expression of *HLA‐DRA* may contribute to a decreased risk of certain other disorders, including hematuria, chronic sinusitis, hyperlipidaemia, and inflammatory spondyloarthropathy. Additionally, no significant adverse effects were identified. In the pathogenesis of sarcopenia, immune cells such as T cells, B cells, and macrophages accumulate in muscle tissue, releasing inflammatory factors that lead to muscle fibre damage and inflammatory responses.^S8^ Therefore, given the important role of HLA‐DRA in immune responses, it may play a role in sarcopenia. Regrettably, aside from HLA‐DRA binding agent *Coccidioides immitis* spherule used for delayed‐type hypersensitivity skin antigen testing, no HLA‐DRA inhibitors are currently applied in clinical settings.^S9^

The *MAP 3K3* gene, also known as mitogen‐activated protein kinase kinase kinase 3, is a human gene that primarily functions by activating the encoded protein involved in the mitogen‐activated protein kinase (MAPK) signalling pathway, which plays a crucial role in cellular signal transduction, particularly in regulating processes such as cell growth, differentiation, and apoptosis.^S10^ Our study found that up‐regulation of *MAP 3K3* is associated with a reduced risk of sarcopenia. However, the mechanism between *MAP 3K3* and sarcopenia is currently unclear. Given the role of the *MAP 3K3* gene in cell growth and differentiation,^S10^ it cannot be entirely ruled out that it may indirectly affect muscle development and maintenance. Although compounds (such as Fostamatinib) associated with the *MAP 3K3* gene have been granted clinical authorization, they function as inhibitors.^S11–S13^ Therefore, there is a prospective need for the development of agonists associated with the *MAP 3K3* gene for the therapeutic intervention of sarcopenia.

*MFGE8* (milk fat globule‐EGF factor 8) is a protein widely expressed in mammals playing crucial roles in various physiological processes, including immune regulation, cellular phagocytosis, and clearance of apoptotic cells.^S14^ Furthermore, MFGE8 promotes the engulfment of apoptotic cells by interacting with phosphatidylserine and αvβ3/αvβ5 integrins, thereby preventing inflammation and autoimmune responses.^S15^ Our study suggests that increased protein abundance or up‐regulation of transcription levels of MFGE8 is associated with a reduced risk of sarcopenia, consistent with previous research on human sarcopenia conditions.^S16^ Animal experiments have indicated that MFGE8 may be involved in the pathogenesis of sarcopenia by modulating cellular apoptosis and oxidative stress responses.^S16–S18^ However, there are currently no drugs developed specifically targeting the *MFGE8* gene. Given its pivotal role in multiple physiological processes, the development of drugs targeting the *MFGE8* gene might offer potential therapeutic advantages for the treatment of sarcopenia. In addition, the expression of *AURKA* in blood and skeletal muscle tissues exhibits an inverse relationship with the risk of sarcopenia. Therefore, further validation in various tissues is warranted to understand the role of *AURKA* better.

Our study has several strengths. Firstly, we directly selected genes that have been confirmed to be related to drug targets, increasing the reliability of our results and the success rate of future drug development. Secondly, we analysed the data from both gene expression and protein expression levels, combined with sarcopenia GWAS datasets, making our findings more persuasive. Importantly, the cis‐acting variants we chose can more easily reveal gene regulatory mechanisms and signalling pathways. Furthermore, our results underwent multiple corrections and Bayesian co‐localization analysis and were cross‐validated across different outcomes and datasets, providing more robust evidence. Lastly, we validated the safety of druggable genes through Phe‐MR analysis, which has guiding implications for subsequent drug development.

Despite some novel findings, our study has potential limitations. Firstly, the absence of comprehensive pQTL data for skeletal muscle within public databases has constrained our ability to perform an exhaustive pQTL analysis on this tissue. We anticipate the future availability of skeletal muscle pQTL datasets, which would allow for more detailed investigations into this area. Secondly, in our MR analysis, to secure potent IVs and mitigate pleiotropy, we mostly used a single SNP as an instrumental variable for our exposure after reducing the impact of genetic linkage disequilibrium using the ‘clump’ function.^S19^ Consequently, we could not perform sensitivity, heterogeneity, and pleiotropy analyses. Future research may consider using more SNPs for a more comprehensive analysis. Thirdly, our selected GWAS databases are based on European populations, which limits its generalizability to other ethnic groups. Additionally, it warrants special attention that MR analyses are designed to explore the associations between genetic variants and the risk of diseases, rather than direct interventional studies.

## Conclusion

Our findings highlight potential targets for future treatment of sarcopenia, necessitating further research to evaluate the feasibility of these five identified druggable genes (*HP*, *HLA‐DRA*, *MAP 3K3*, *MFGE8*, and *COL15A1*) as therapeutic drug targets for sarcopenia, especially for zinc supplementation and collagenase *Clostridium histolyticum* , which target for HP and COL15A1, respectively. The role of action for *AURKA* necessitates further validation in various tissues for a comprehensive understanding. Nevertheless, caution must be exercised when considering therapeutic recommendations derived from the results of MR analyses, as they necessitate validation through meticulously executed clinical trials.

## Conflict of interest

The authors declare that there is no conflict of interest.

## Funding

This study was supported by the National Natural Science Fund of Sichuan (Grant no. 2022NSFSC0749 to B.C.), the 135 Project for Disciplines of Excellence–Clinical Research Fund, West China Hospital, Sichuan University (Grant No. 2023HXFH032 to Y.P.C.), the National Key Research and Development Program of China (Grant no. 2022YFC2703101 to Y.P.C.), the Science and Technology Bureau Fund of Sichuan Province (Grant no 2023YFS0269 to Y.P.C.), and the National Natural Science Fund of China (Grant No. 81971188 to Y.P.C. and Grant No. 82371422 to Y.P.C.).

## Supporting information


**Figure S1.** Manhattan and Venn plots of preliminary MR analysis for druggable genes associated with blood and skeletal muscle after Bonferroni correction (at least in two outcomes or datasets). **Figure S1A** shows that in the blood pQTL dataset, the *HP* gene passed the Bonferroni correction and is present in two outcomes simultaneously. **Figure S1B** shows that in the blood eQTL dataset, the *EHMT2* gene passed the Bonferroni correction and is present in two outcomes simultaneously. **Figure S1C** shows that in the skeletal muscle eQTL dataset, four druggable genes passed the Bonferroni correction and are present in two outcomes simultaneously. **Figure S1D** shows that 11 druggable genes passed the Bonferroni correction and are present in two datasets simultaneously. ALM, appendicular lean mass.
**Figure S2.** Protein–protein interaction (PPI) networks for druggable genes were analysed both pre‐ and post‐Bayesian analysis. **Figure S2A** shows that PPI results for the 17 druggable genes identified prior to Bayesian analysis revealed interactions among certain genes. The PPI network comprised 17 nodes, representing 17 unique proteins, with 12 edges indicating the number of interactions between proteins. Notably, CTSS and CTNNB1 were central in the network. **Figure S2B** shows that PPI analysis was conducted on the 6 druggable genes identified following Bayesian analysis. However, this analysis did not demonstrate any interactions among these genes.
**Figure S3.** Phenome‐Wide MR analysis identified the potential side effects or additional indications of six prior druggable genes for sarcopenia. We found that only 2 druggable genes (*HP* and *HLA‐DRA*) have associated additional indications (9 SNPs for the 6 druggable genes were used for Phe‐MR analysis, P‐value < 0.05/9/784), while no side effects were discovered for the 6 druggable genes. Upregulation of *HP* expression and suppression of *HLA‐DRA* expression can reduce the risk of sarcopenia and also have beneficial effects on dyslipidemia, hematuria, chronic sinusitis, and inflammatory spondylopathy.

**Table S1.** Druggable genes from the Drug‐Gene Interaction Database (DGIdb).
**Table S2.** Druggable genes from the study by Finan et al.
**Table S3.** Overlapping druggable genes used in this study.
**Table S4.** After filtering, the instrumental variables of druggable genes used for this study from the three datasets.
**Table S5.** MR analysis of druggable genes and sarcopenia in blood pQTL datasets (P value < 0.05).
**Table S6.** MR analysis of druggable genes and sarcopenia in blood eQTL datasets (P value < 0.05).
**Table S7.** MR analysis of druggable genes and sarcopenia in muscle eQTL datasets (P value < 0.05).
**Table S8.** MR analysis of druggable genes and sarcopenia in blood pQTL datasets (Bonferroni‐corrected P value < 0.05/187).
**Table S9.** MR analysis of druggable genes and sarcopenia in blood eQTL datasets (Bonferroni‐corrected P value < 0.05/1531).
**Table S10.** MR analysis of druggable genes and sarcopenia in muscle eQTL datasets (Bonferroni‐corrected P value < 0.05/419).
**Table S11.** Summary findings, including bonferroni correction, colocalization and sensitivity analysis.
**Table S12.** UKBiobank phenotypes for Phe‐MR analysis.
**Table S13.** Phenome‐Wide MR analysis identified the potential side effects or additional indications of prior druggable genes for sarcopenia.

## Data Availability

Any data generated in the analysis process can be requested from the corresponding author.
