# Supplementary material for: Systematic druggable genome‐wide Mendelian randomization identifies therapeutic targets for sarcopenia
Source: J Cachexia Sarcopenia Muscle. 2024 Apr 21;15(4):1324–34. doi: 10.1002/jcsm.13479 (PMC11294052; doi:10.1002/jcsm.13479)
Supplement: Supplementary file 1 — Figure S1. Manhattan and Venn plots of preliminary MR analysis for druggable genes associated with blood and skeletal muscle after Bonferroni correction (at least in two outcomes or datasets). Figure S1A shows that in the blood pQTL dataset, the HP gene passed the Bonferroni correction and is present in two outcomes simultaneously. Figure S1B shows that in the blood eQTL dataset, the EHMT2 gene passed the Bonferroni correction and is present in two outcomes simultaneously. Figure S1C shows that in the skeletal muscle eQTL dataset, four druggable genes passed the Bonferroni correction and are present in two outcomes simultaneously. Figure S1D shows that 11 druggable genes passed the Bonferroni correction and are present in two datasets simultaneously. ALM, appendicular lean mass. Figure S2. Protein–protein interaction (PPI) networks for druggable genes were analysed both pre‐ and post‐Bayesian analysis. Figure S2A shows that PPI results for the 17 druggable genes identified prior to Bayesian analysis revealed interactions among certain genes. The PPI network comprised 17 nodes, representing 17 unique proteins, with 12 edges indicating the number of interactions between proteins. Notably, CTSS and CTNNB1 were central in the network. Figure S2B shows that PPI analysis was conducted on the 6 druggable genes identified following Bayesian analysis. However, this analysis did not demonstrate any interactions among these genes. Figure S3. Phenome‐Wide MR analysis identified the potential side effects or additional indications of six prior druggable genes for sarcopenia. We found that only 2 druggable genes (HP and HLA‐DRA) have associated additional indications (9 SNPs for the 6 druggable genes were used for Phe‐MR analysis, P‐value < 0.05/9/784), while no side effects were discovered for the 6 druggable genes. Upregulation of HP expression and suppression of HLA‐DRA expression can reduce the risk of sarcopenia and also have beneficial effects on dyslipidemia, hematuria, ch [file JCSM-15-1324-s001.docx]

**Systematic druggable genome-wide Mendelian randomization identifies therapeutic targets for sarcopenia**

Kang-Fu Yin^1, 2, #^, Ting Chen^1, 2, #^, Xiao-Jing Gu^3^, Wei-Ming Su^1, 2^, Zheng Jiang^1, 2^, Si-Jia Lu^4^, Bei Cao^1, 2^, Li-Yi Chi^5^, Xia Gao^6, *^, Yong-Ping Chen^1, 2, *^

**Supplementary references**

S1. Buehler PW, Humar R, Schaer DJ. Haptoglobin Therapeutics and Compartmentalization of Cell-Free Hemoglobin Toxicity. *Trends In Molecular Medicine* 2020;26(7):683-97.

S2. Nascimento CM, Ingles M, Salvador-Pascual A, Cominetti MR, Gomez-Cabrera MC, Viña J, et al. Sarcopenia, frailty and their prevention by exercise. *Free radical biology & medicine* 2019;132:42-49.

S3. Zou Y, Zwolanek D, Izu Y, Gandhy S, Schreiber G, Brockmann K, et al. Recessive and dominant mutations in COL12A1 cause a novel EDS/myopathy overlap syndrome in humans and mice. *Human Molecular Genetics* 2014;23(9):2339-52.

S4. Hicks D, Farsani GT, Laval S, Collins J, Sarkozy A, Martoni E, et al. Mutations in the collagen XII gene define a new form of extracellular matrix-related myopathy. *Human Molecular Genetics* 2014;23(9):2353-63.

S5. Shiina T, Hosomichi K, Inoko H, Kulski JK. The HLA genomic loci map: expression, interaction, diversity and disease. *Journal of Human Genetics* 2009;54(1):15-39.

S6. Castro F, Acevedo E, Ciusani E, Angulo JA, Wollheim FA, Sandberg-Wollheim M. Tumour necrosis factor microsatellites and HLA-DRB1*, HLA-DQA1*, and HLA-DQB1* alleles in Peruvian patients with rheumatoid arthritis. *Annals of the Rheumatic Diseases* 2001;60(8):791-95.

S7. Zhou X-J, Cheng F-J, Zhu L, Lv JC, Qi YY, Hou P, et al. Association of systemic lupus erythematosus susceptibility genes with IgA nephropathy in a Chinese cohort. *Clinical journal of the American Society of Nephrology : CJASN* 2014;9(4):788-97.

S8. Gan L, Miller FW. State of the art: what we know about infectious agents and myositis. *Current opinion in rheumatology* 2011;23(6):585-94.

S9. Calhoun DL, Osir EO, Dugger KO, Galgiani JN, Law JH. Humoral antibody responses to specific antigens of Coccidioides immitis. *The Journal of infectious diseases* 1986;154(2):265-72.

S10. Cargnello M, Roux PP. Activation and function of the MAPKs and their substrates, the MAPK-activated protein kinases. *Microbiol Mol Biol Rev* 2011;75(1):50-83.

S11. Wigerblad G, Warner SA, Ramos-Benitez MJ, Kardava L, Tian X, Miao R, et al. Spleen tyrosine kinase inhibition restores myeloid homeostasis in COVID-19. *Science Advances* 2023;9(1):eade8272.

S12. Bussel J, Arnold DM, Grossbard E, Mayer J, Treliński J, Homenda W, et al. Fostamatinib for the treatment of adult persistent and chronic immune thrombocytopenia: Results of two phase 3, randomized, placebo-controlled trials. *American Journal of Hematology* 2018;93(7):921-30.

S13. Weinblatt ME, Genovese MC, Ho M, Hollis S, Rosiak-Jedrychowicz K, Kavanaugh A, et al. Effects of fostamatinib, an oral spleen tyrosine kinase inhibitor, in rheumatoid arthritis patients with an inadequate response to methotrexate: results from a phase III, multicenter, randomized, double-blind, placebo-controlled, parallel-group study. *Arthritis Rheumatol* 2014;66(12):3255-64.

S14. Hanayama R, Tanaka M, Miwa K, Shinohara A, Iwamatsu A, Nagata S. Identification of a factor that links apoptotic cells to phagocytes. *Nature* 2002;417(6885):182-87.

S15. Aziz M, Jacob A, Matsuda A, Wang P. Review: milk fat globule-EGF factor 8 expression, function and plausible signal transduction in resolving inflammation. *Apoptosis : an International Journal On Programmed Cell Death* 2011;16(11):1077-86.

S16. Li H, Guan K, Liu D, Liu M. Identification of mitochondria-related hub genes in sarcopenia and functional regulation of MFG-E8 on ROS-mediated mitochondrial dysfunction and cell cycle arrest. Food Funct. 2022 Jan 24;13(2):624-638. PMID: 34928287.

S17. Guan K , Li H , Chen H , Qi X , Wang R , Ma Y . TMT-based quantitative proteomics analysis reveals the effect of bovine derived MFG-E8 against oxidative stress on rat L6 cells. Food Funct. 2021 Aug 21;12(16):7310-7320. PMID: 34169949.

S18. Li H, Guan K, Wang R, Zhu A, Ma Y. Synergistic effects of MFG-E8 and whey protein on mitigating d-galactose-induced sarcopenia through PI3K/AKT/PGC-1α and MAPK/ERK signaling pathways. J Dairy Sci. 2024 Jan;107(1):9-23. PMID: 37678791.

S19. Hemani G, Zheng J, Elsworth B, Wade KH, Haberland V, Baird D, et al. The MR-Base platform supports systematic causal inference across the human phenome. *ELife* 2018;7.

S20. Rolf MG, Curwen JO, Veldman-Jones M, Eberlein C, Wang J, Harmer A, Hellawell CJ, Braddock M. In vitro pharmacological profiling of R406 identifies molecular targets underlying the clinical effects of fostamatinib. Pharmacol Res Perspect. 2015 Oct;3(5):e00175. PMID: 26516587; PMCID: PMC4618646.

S21. Hun-Chi Lin, Shau-Ping Lei, "Molecular cloning of the genes responsible for collagenase production from Clostridium histolyticum." U.S. Patent US5177017, issued December, 1972. [US5177017](https://www.google.com/?tbm=pts#q=5177017&tbm=pts).

S22. de Groot CO, Hsia JE, Anzola JV, Motamedi A, Yoon M, Wong YL, Jenkins D, Lee HJ, Martinez MB, Davis RL, Gahman TC, Desai A, Shiau AK. A Cell Biologist's Field Guide to Aurora Kinase Inhibitors. Front Oncol. 2015 Dec 21;5:285. PMID: 26732741; PMCID: PMC4685510.

S23. Gilman JG, Brewer GJ. The oxygen-linked zinc-binding site of human haemoglobin. Biochem J. 1978 Mar 1;169(3):625-32. PMID: 646791; PMCID: PMC1183835.

S24. Carlo-Stella C, Di Nicola M, Turco MC, Cleris L, Lavazza C, Longoni P, Milanesi M, Magni M, Ammirante M, Leone A, Nagy Z, Gioffrè WR, Formelli F, Gianni AM. The anti-human leukocyte antigen-DR monoclonal antibody 1D09C3 activates the mitochondrial cell death pathway and exerts a potent antitumor activity in lymphoma-bearing nonobese diabetic/severe combined immunodeficient mice. Cancer Res. 2006 Feb 1;66(3):1799-808. PMID: 16452241.

S25. Wieczorek M, Abualrous ET, Sticht J, Álvaro-Benito M, Stolzenberg S, Noé F, Freund C. Major Histocompatibility Complex (MHC) Class I and MHC Class II Proteins: Conformational Plasticity in Antigen Presentation. Front Immunol. 2017 Mar 17;8:292. PMID: 28367149; PMCID: PMC5355494.


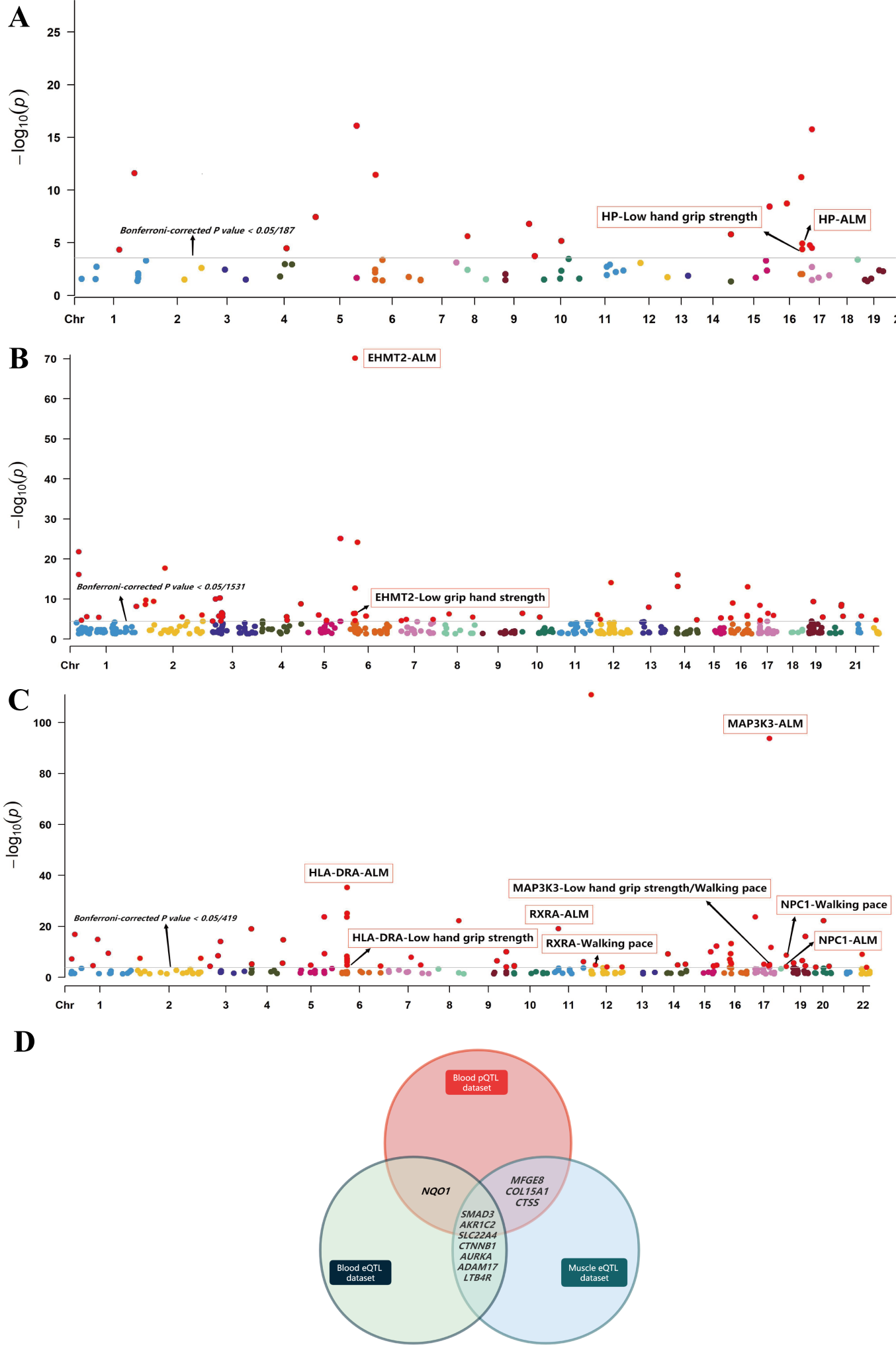


**Figure S1.** Manhattan and Venn plots of preliminary MR analysis for druggable genes associated with blood and skeletal muscle after Bonferroni correction (at least in two outcomes or datasets). **Figure S1A** shows that in the blood pQTL dataset, the *HP* gene passed the Bonferroni correction and is present in two outcomes simultaneously. **Figure S1B** shows that in the blood eQTL dataset, the *EHMT2* gene passed the Bonferroni correction and is present in two outcomes simultaneously. **Figure S1C** shows that in the skeletal muscle eQTL dataset, four druggable genes passed the Bonferroni correction and are present in two outcomes simultaneously. **Figure S1D** shows that 11 druggable genes passed the Bonferroni correction and are present in two datasets simultaneously. ALM, appendicular lean mass.


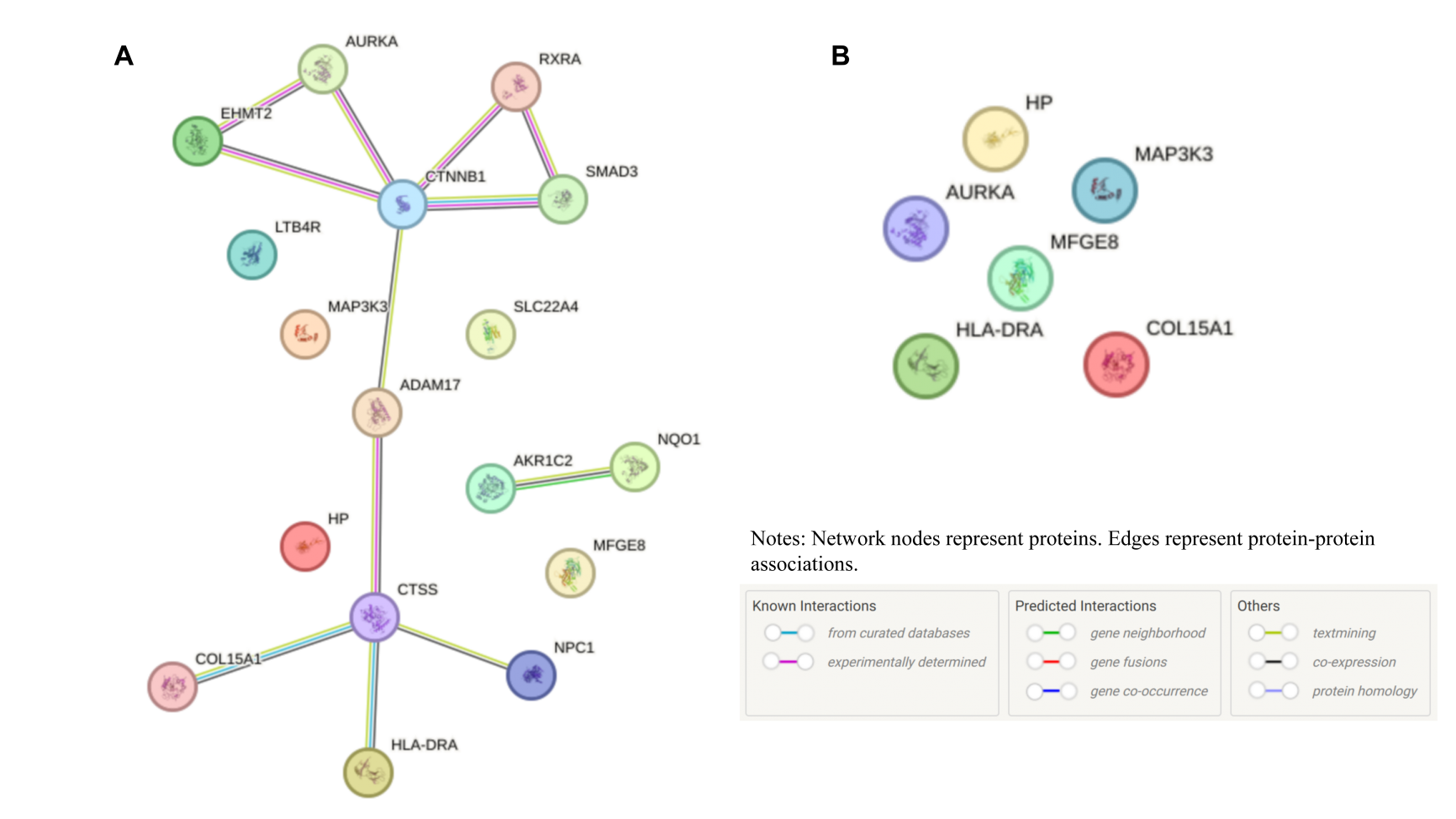


**Figure S2.** Protein-protein interaction (PPI) networks for druggable genes were analyzed both pre- and post-Bayesian analysis. **Figure S2A** shows that PPI results for the 17 druggable genes identified prior to Bayesian analysis revealed interactions among certain genes. The PPI network comprised 17 nodes, representing 17 unique proteins, with 12 edges indicating the number of interactions between proteins. Notably, CTSS and CTNNB1 were central in the network. **Figure S2B** shows that PPI analysis was conducted on the 6 druggable genes identified following Bayesian analysis. However, this analysis did not demonstrate any interactions among these genes.


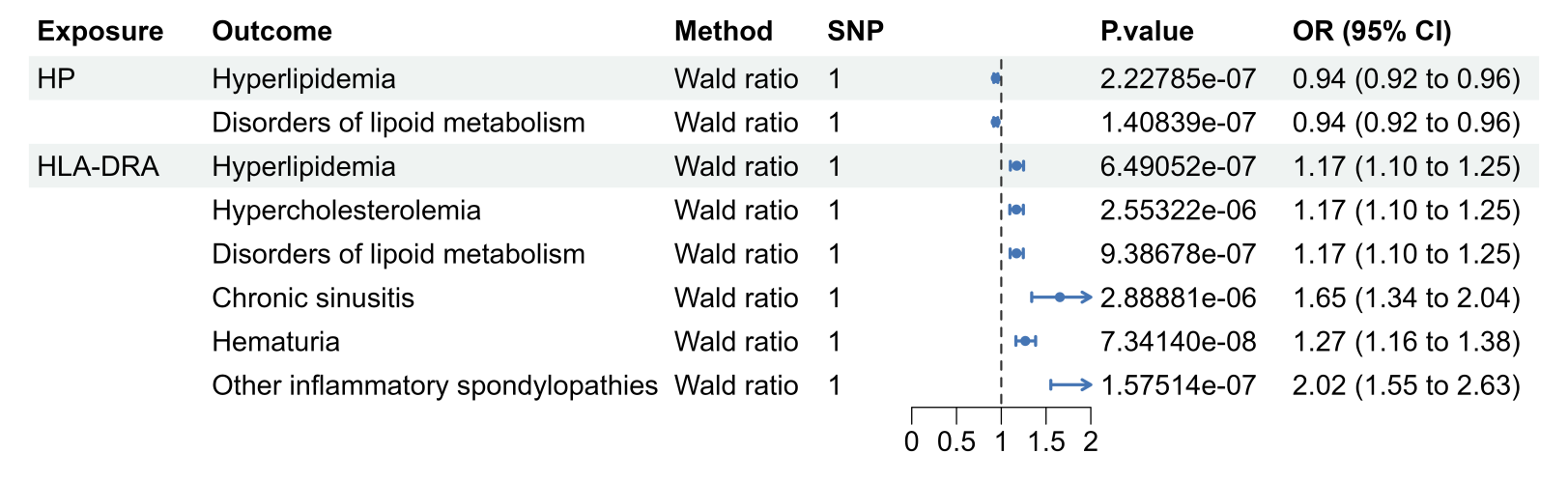


**Figure S3.** Phenome-Wide MR analysis identified the potential side effects or additional indications of six prior druggable genes for sarcopenia. We found that only 2 druggable genes (*HP* and *HLA-DRA*) have associated additional indications (9 SNPs for the 6 druggable genes were used for Phe-MR analysis, P-value < 0.05/9/784), while no side effects were discovered for the 6 druggable genes. Upregulation of *HP* expression and suppression of *HLA-DRA* expression can reduce the risk of sarcopenia and also have beneficial effects on dyslipidemia, hematuria, chronic sinusitis, and inflammatory spondylopathy.
